# CNN-Peaks: ChIP-Seq peak detection pipeline using convolutional neural networks that imitate human visual inspection

**DOI:** 10.1038/s41598-020-64655-4

**Published:** 2020-05-13

**Authors:** Dongpin Oh, J. Seth Strattan, Junho K. Hur, José Bento, Alexander Eckehart Urban, Giltae Song, J. Michael Cherry

**Affiliations:** 1 School of Computer Science and Engineering, Pusan National University, Busan, 46241 South Korea; 2 Department of Genetics, Stanford University, Stanford, 94305 USA; 3 School of Medicine, Kyung Hee University, Seoul, 02447 South Korea; 4 Department of Computer Science, Boston College, Chestnut Hill, Philadelphia, MA 02467 USA

**Keywords:** Computational biology and bioinformatics, Genetics, Molecular biology

## Abstract

ChIP-seq is one of the core experimental resources available to understand genome-wide epigenetic interactions and identify the functional elements associated with diseases. The analysis of ChIP-seq data is important but poses a difficult computational challenge, due to the presence of irregular noise and bias on various levels. Although many peak-calling methods have been developed, the current computational tools still require, in some cases, human manual inspection using data visualization. However, the huge volumes of ChIP-seq data make it almost impossible for human researchers to manually uncover all the peaks. Recently developed convolutional neural networks (CNN), which are capable of achieving human-like classification accuracy, can be applied to this challenging problem. In this study, we design a novel supervised learning approach for identifying ChIP-seq peaks using CNNs, and integrate it into a software pipeline called CNN-Peaks. We use data labeled by human researchers who annotate the presence or absence of peaks in some genomic segments, as training data for our model. The trained model is then applied to predict peaks in previously unseen genomic segments from multiple ChIP-seq datasets including benchmark datasets commonly used for validation of peak calling methods. We observe a performance superior to that of previous methods.

## Introduction

In the postgenomic era, understanding epigenetic regulation is one of the most important challenges in the biological and medical sciences. To elucidate pathological mechanisms, and pinpoint target genes for developing therapeutics, it is important to assess abnormalities in the genome-wide interactions of proteins and genomic elements that cause gene misregulation. In this regard, chromatin immunoprecipitation followed by sequencing (ChIP-seq) is a technique widely used to identify genomic binding sites for epigenetic regulators, including histones, transcription factors, and DNA/RNA binding proteins^1,2^. ChIP-seq enables the discovery of the interactions between protein complexes and DNA regulatory elements, and their gene regulatory networks^3^. ChIP-seq data analysis has shown how histone modifications and nucleic acid interacting proteins modulate the critical factors of gene regulation, and cell lineage determination and maintenance^4,5^.

One of the major computational challenges in analyzing ChIP-seq data is to identify peaks in genomic areas where aligned reads are enriched when sequencing reads are mapped to a given reference genome. This task is a challenge for several reasons. Sequencing errors and local bias caused by structural variations complicate solving the peak-calling problem. The diverse patterns of data, which are caused by biological variability, a variety of domains, experiment environment, and sequencing coverage, make the problem even harder. Sensitive and reliable computational methods to determine peaks from noisy backgrounds are especially important for medical studies, where patient samples can be limited, resulting in sub-optimal data quantity and quality.

Several software tools for calling the peaks in ChIP-seq data have been developed based on various probabilistic and unsupervised learning methods, such as MACS2, HOMER, SICER, and SPP^4,6,7^. Some of these tools show the high sensitivity of calling the peaks, but suffer from high false-positive error rates. Other tools may need additional information as input data (e.g. mappability scores that the fraction of a region that overlaps at least one uniquely mappable read in the genome^8^).

In^9^ a neural network model was proposed to denoise ChIP-seq data and increase the performance of calling the peaks. An ensemble approach was also proposed^10^ to improve the accuracy of calling ChIP-seq peaks. The latter exploits the output from multiple existing peak-calling software tools to eliminate outliers among the multiple peak calling decisions. These approaches have increased the sensitivity of calling true peaks, but they still suffer from high false-positive rates^8^. In particular, human cancer cell lines are too complicated for one to understand human malignancy using ChIP-seq, since the data patterns vary substantially depending on patients’ primary cancer tissues^11–13^. False-positive rates of calling ChIP-seq peaks in human cancer cell lines are worse than in other datasets^12^.

To resolve these issues, human experts are used to label true peaks using visualization tools such as UCSC genome browser, and the Integrative Genomics Viewer (IGV)^14,15^. False-positive peaks can also be corrected by professional researchers^16^. However, it would be extremely inefficient for human scientists to find all of the peaks in the whole genome for large volumes of ChIP-seq data. Hocking *et al*.^8^, have proposed a supervised learning approach based on grid search to optimize a parameter (e.g. a cut-off value) that users need to set up when running peak-calling tools such as MACS2. They use data of which part of the peaks have been labeled by human experts, learn parameter values, and apply the optimized parameters for the rest of the dataset. Unfortunatly, this requires completing labeling task for each individual ChIP-seq dataset and each peak-calling tool.

Recently, supervised machine learning methods based on deep neural networks, such as convolutional neural networks (CNN), have been successfully applied to epigenetics, regulatory genomics, and system biology^17,18^. In this article, we develop a novel peak calling software pipeline based on CNN, named CNN-Peaks, that feeds on data partially labeled by human researchers. We identify local peak-calling threshold values, which might differ from one genomic segment to another, and use them to reduce false-positive peak calls, and thus resolve local bias caused by structural variations in, e.g., the human cancer cell lines. Note that some of the peaks in genomic regions that have frequent copy number variations are expected to show higher mapping depth than the others. As training data for CNN-Peaks, we use data labeled by human experts as well as read count information from the preprocessing steps of raw read mapping. Our software tool learns a model for determining proper peak-detection cut-off values in specific genomic regions by taking read mapping patterns in their neighbor regions into account. Our inspiration to use CNNs is the fact that they have been successfully used to solve problems in image processing and natural language translation using data patterns that, like in our case, have features with local dependencies^19,20^). We also use integrated annotation information (RefSeq) available in NCBI (https://www.ncbi.nlm.nih.gov/) as training input data for building our predictive CNN. The RefSeq data include genomic locations of genes, transcripts and protein encodings that can assist to determine the existence of peaks for human visual inspection.

Our CNN-Peaks software package is composed of three main modules: one for preprocessing original input data (labeled data, genome annotation, and read count information) and feed them into our CNN architecture; another for learning a model using the training data; and a final one for predicting peaks for unknown data. We evaluate the performance of CNN-Peaks using some labeled data reserved as test data. We also test CNN-Peaks on ChIP-seq benchmark datasets that are commonly used for evaluation, and compare its performance against other major peak calling tools. We also use our CNN-Peaks tool to analyze various real datasets for histone modification, such as H3K27ac, and for transcription factor binding, such as GATAD2, in various human cell lines.

Users can install the CNN-Peaks pipeline using a docker image and run the package using their own data with our trained model for predicting narrow histone modifications and transcription factor binding sites in humans. Experienced users can also build a new predictive model trained on their own labeled data using CNN-Peaks. Our software package includes a desktop application to help experts create labeled data easily by labeling peaks in genomic regions randomly selected from a given ChIP-seq read mapping data (Supplementary text S3). Our pipeline can be also applied to other types of high-throughput sequencing data, such as DNase-seq and ATAC-seq. Our package is available through the Github repository http://github.com/odb9402/CNNPeaks.

## Materials and Methods

### Data description

**Figure 1:**
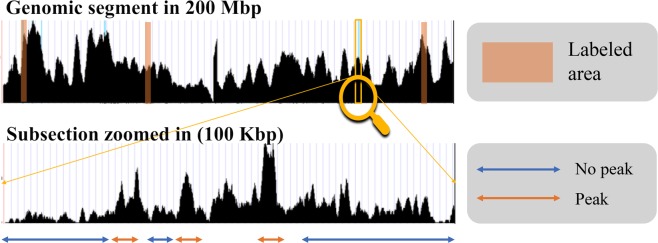
Visualization of labeling peaks using ChIP-seq read mapping data. For some genomic regions, human researchers can determine which regions are peaks, or not, and label them using an interface tool included in our CNN-Peaks package.

We obtained multiple ChIP-seq read mapping datasets in BAM format from the ENCODE data portal^21^ for creating labeled data, and validating CNN-Peaks. This data includes several ChIP-seq datasets for examining histone modification, such as histones H3K36me3, H3K4me3, H3K27me3, H2AFZ, and H3K9ac, and transcription factor binding, such as transcription factors GATAD2, POLR2A, SMARCE1, and MAX in cancer cell domains K562, A549, HepG2, HEK293, and GM12878. We also downloaded ChIP-seq data from leukemia cell line K562 and labeled its background binding of genomic regions to then train our CNN model. From the ChIP-seq read mapping data in BAM format, some genomic segments were randomly selected, and the locations of peaks were labeled using our visualization tool with the BAM alignment^8^ data as Fig. 1 illustrates.

In addition to the labeled data and read mapping BAM files, we used a curated non-redundant collection of genomic, transcript, and protein sequence records for the human reference sequence (RefSeq) in NCBI, as an additional input vector for our CNN model^22^. The RefSeq data include protein-coding locations and pseudogenes. When researchers manually examine ChIP-seq data to determine the peaks, the genome annotation information, such as transcripts and their corresponding protein records, are commonly viewed together with the given read mapping data. This information often gives a good guess of where peaks might be. To mimic human inspection, we considered this and built our model so that it uses these different annotated types as input. We added this annotation information as a binary vector to represent the presence and absence of transcripts and proteins in each genomic position.

### Data preprocessing

A preprocessing module is included in our CNN-Peaks pipeline. This module converts all input data, including read mapping BAM files, labeled data, and genome annotation information, to vectors with the right shape to be fed into our CNN architecture. We use bins of a fixed size (12,000 bins by default) to normalize the different window sizes of labeled segments into the same size. If a window is smaller than the target window size, the user of CNN-Peaks should either label an additional region to append to this window using our visual inspection tool, or should adjust the parameter of the target window size, or should simply eliminate the window (Supplementary text S1).

Our preprocessing module also includes functions to smooth read count patterns to reduce noises in read alignment data. This smoothing step also helps make our CNN model close to human visual inspection. Without the smoothing and correction step, (raw) depth patterns are very noisy (Supplementary text S1). We use max-pooling and Gaussian filter convolution operations for smoothing. Gaussian filters are commonly used for denoising and smoothing in various domains, such as image processing^23^. The equations of the convolution and max-pooling operations (Eqs. (1) and (2) respectively) are as follows (note “*” indicates a convolution operation. The strides for convolution and max-pooling are 1).

1 $$(v\ast u)(n)=\mathop{\sum }\limits_{m=-[\frac{M}{2}]}^{\left[\frac{M}{2}\right]}{v}_{n-m\cdot }{u}_{n}$$

2 $${\max }\,{\_}{poo}{{l}}_{{v}{,}{w}}({n}){=}{{v}}_{{k}}\,\mathrm{where}\,{{\boldsymbol{v}}}_{{\boldsymbol{k}}}\ge {{\boldsymbol{v}}}_{{\boldsymbol{i}}}\,\mathrm{for}\,\mathrm{all}\,{\boldsymbol{n}}-\frac{{\boldsymbol{w}}}{2}\le {\boldsymbol{i}}\le {\boldsymbol{n}}+\frac{{\boldsymbol{w}}}{2}$$

Above, $M,w\in {Z}^{+}$ are the filter size of each operation, *v*, *u* are input vectors, and ${v}_{i}$ is the *i* ^th^ component of $v$. Let $X$ be a vector of read mapping counts, $filter$ a gaussian filter, and ${X}_{smoothi{\rm{ng}}}$ a smoothed vector of $X$, then

3 $${X}_{pool}=[max\_poo{l}_{X,w}(1),\ldots ,max\_poo{l}_{X,w}(L)],$$

4 $${{\boldsymbol{X}}}_{{\boldsymbol{smoothing}}}=[({{\boldsymbol{X}}}_{{\boldsymbol{pool}}}\,\ast \,{\boldsymbol{filter}})(1),\ldots ,({{\boldsymbol{X}}}_{{\boldsymbol{pool}}}\,\ast \,{\boldsymbol{filter}})({\boldsymbol{L}})].$$

### CNN architecture

**Figure 2:**
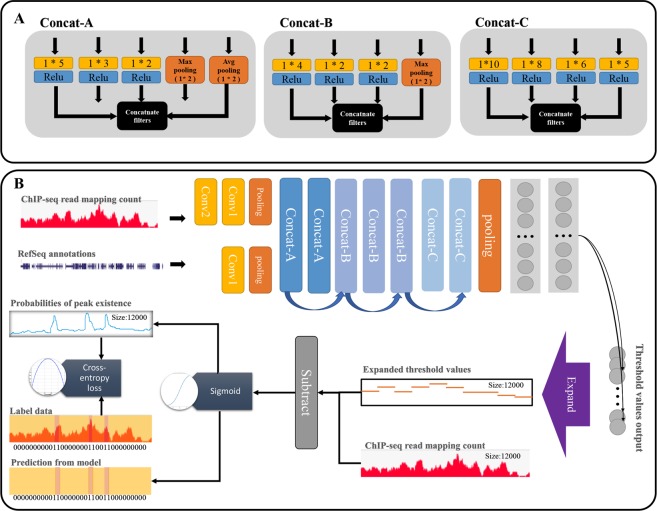
The schema of CNN-peaks model. (**A**) There are three Inception-like modules: Concat-A, Concat-B, and Concat-C. Each yellow box represents a convolution operation with filter size 1 * N, and black boxes concatenate filters, which are constructed by combining several convolution and pooling outputs. (**B**) Our model learns optimal threshold values for calling peaks in local genomic segments of ChIP-seq data and determines the presence or absence of peaks using a subtraction of the threshold from each input signal value, and a sigmoid operation. Blue arrows indicate residual connections between Inception modules, and a purple arrow an operation for expanding output vectors.

Our CNN architecture builds upon the Inception module used in GoogLeNet, which extracts diversified features from data via filters of various sizes (see various filters in Fig. 2A)^24,25^. We built three types of modules with a slimmer structure than the original Inception module as Fig. 2B illustrates. The structure is composed of several hidden layers, such as pooling and convolutional layers, as well as the Inception modules, as Fig. 2 shows. The Inception-style modules are known to lead to inefficacies in terms of prediction accuracy if they are placed at the beginning of the CNN architecture^24^. To avoid this potential problem, we put traditional convolutional filters, and a max-pooling layer, at the beginning of our CNN-Peaks architecture (Fig. 2). The details of our CNN architecture are described as follows.

First, two input vectors for annotation information (RefSeq) and read mapping patterns are transformed through the convolution layers into vectors. Then the max-pooling layer reduces the dimension of these vectors. Then there are seven Inception modules. Each Inception module has a layer that concatenates a list of outputs from the filters. Two of these modules, called A-modules, use three types of filters: convolution, max, and average pooling. They provide information about peak patterns and the scale of strong peak signals to our CNN model. However, since max pooling and average pooling have the same number of features as the previous layer, this increases the vector size exponentially as a function of the depth of pooling layers^24^. To resolve this issue, we use the other three Inception modules, called B-modules, which are composed of convolution filters of three types without an average pooling. Finally, we use two Inception modules, called C-modules, that have four types of convolution filters with a wider size and longer stride than the filters used in A- and B-modules. At the end of the Inception modules, an average pooling layer followed by fully connected layers is added to reduce dimensions.

In addition, we use a *residual structure* between the Inception layers^26^. This helps avoiding vanishing gradient problems. We also use batch normalization as regularization to avoid overfitting^27^ while training.

### Output layer of CNN architecture

**Figure 3:**
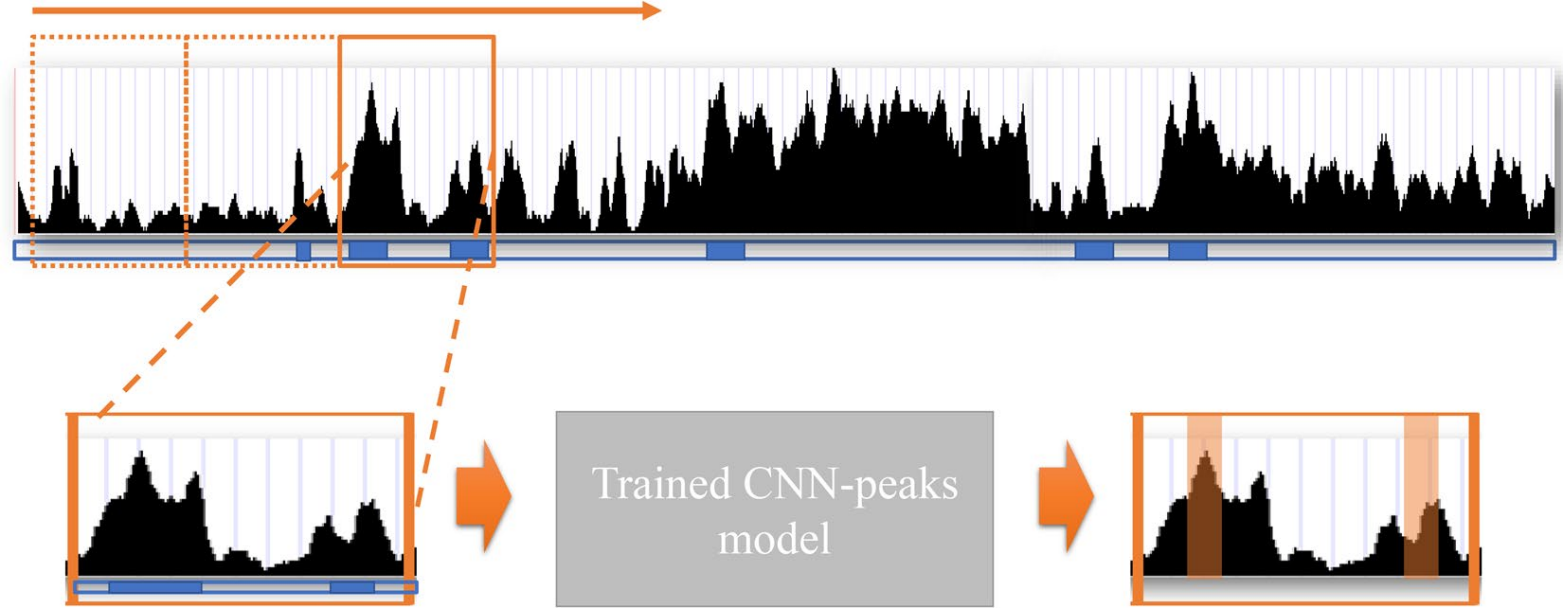
The process of peak calling with a trained model. The black signal is the read mapping depth in the ChIP-seq input data, and the blue boxes below the signal indicate the presence of genes in RefSeq annotation. An orange box is a window with both read mapping signal and RefSeq annotation in a genomic region. Peaks (in orange underlay) in the window are predicted using the model trained by CNN-Peaks, and generated in BED format.

To determine the presence or absence of peaks in each individual genomic position, the output layer of the CNN architecture needs a number of neurons that are equal to the number of genome bases. This large number of neurons in the output layer usually causes a significant degradation of learning performance^28^. In order to reduce the number of neurons, we designed our CNN model to learn optimal threshold values for genomic segments based on read mapping patterns in a selected window, rather than computing the p-value or the likelihood of the presence of a peak signal in each individual genomic position. This substantially reduces the number of neurons required in the output layer of our CNN model, and prevents performance degradation. Since the output vector size becomes smaller than the input vector, we add an additional operation to expand the output vector size to be identical to the input vector, so that we can predict the presence or absence of peaks in each individual position (See the purple box named as “Expand” in Fig. 2B). These expanding vectors are implemented using the “broadcasting vector” standard in Tensorflow and Numpy, which allows operations between vectors of different sizes. The peak calling process of our CNN-Peaks is summarized in Fig. 3.

### Loss function

Determining the presence or absence of a peak signal is a binary classification problem. We use cross-entropy as a loss function for learning our model. Most methods for classification problems require balancing the trade-off between sensitivity and specificity in performance. Likewise, we need to be careful not to favor only one of these^29^. In peak calling problems with ChIP-seq data, peaks are relatively rare compared to the whole genome size. If a certain method tends to call ‘no-peak’ (*i.e*. it has low false-positive error rate), it will show high accuracy, although it misses important peaks. Therefore, we use a weighted cross-entropy as our loss, in Eq. (5).

5 $${\ell }_{{\boldsymbol{i}}}({\boldsymbol{X}},{\boldsymbol{\Lambda }},{\boldsymbol{\theta }})={{\boldsymbol{y}}}_{{\boldsymbol{i}}}\,\log ({{\boldsymbol{h}}}_{{\boldsymbol{i}},{\boldsymbol{\theta }}}({\boldsymbol{X}},{\boldsymbol{\Lambda }})){\boldsymbol{w}}+(1-{{\boldsymbol{y}}}_{{\boldsymbol{i}}})\log (1-{{\boldsymbol{h}}}_{{\boldsymbol{i}},{\boldsymbol{\theta }}}({\boldsymbol{X}},{\boldsymbol{\Lambda }})),$$

where *X* is the input read counts vector, $\varLambda$ the annotation vector, $\theta$ the set of parameters in our model, ${y}_{i}$ the *i*^th^ element of the input for the labeled data, $w\,$ a weight for the importance of false-negative calls relative to false-positives calls in the valuation, and ${h}_{i,{\rm{\theta }}}(X,\Lambda )$ is the *i*^th^ element in the output predicted for given input $X$, $\Lambda \,$ and model parameter $\theta .$ The weight $w$ is determined by a ratio between negative regions (no peaks) and positive regions (peaks) for given data.

In addition, we apply the Top-*K* method for the loss function^30^. In the Top-*K* method, sensitivity is regarded as more important than specificity for a high value of *K*, while specificity is more important than sensitivity for a low value of *K*. To achieve a balance between sensitivity and specificity, we set *L* = *K*/2, where *L* is the output vector size. Our final loss function is (6)

6 $${{\boldsymbol{ {\mathcal L} }}}_{{\boldsymbol{TopK}}}({\boldsymbol{X}},{\boldsymbol{\Lambda }},{\boldsymbol{\theta }},{\boldsymbol{\kappa }})=\frac{1}{{\boldsymbol{\kappa }}}\mathop{\sum }\limits_{{\boldsymbol{n}}=1}^{{\boldsymbol{\kappa }}}{\ell }_{[{\boldsymbol{n}}]}({\boldsymbol{X}},{\boldsymbol{\Lambda }},{\boldsymbol{\theta }}),$$

where ${\ell }_{[n]}(X,\Lambda ,\theta )$ is the $n$ ^th^ largest individual weighted cross-entropy loss among all ${l}_{i}$. The loss function ${ {\mathcal L} }_{TopK}$ was optimized using the Adam optimizer that uses backpropagation to adjust model the parameters $\theta$^31^.

### Scoring peaks using sigmoid activations

An important task of any peak-calling algorithm is to give a significance score to each peak^32^. We measure the significance of peaks by calculating a *p*-value for each genomic location under the Poisson distribution (note that the counts for each position in the genome-wide tag data for ChIP experiments is known to follow a Poisson distribution)^32,33^ and by combining these *p*-values with the sigmoid values from the output layer in the CNN-Peaks architecture. The sigmoid function in the output layer of our CNN-Peaks model generates values that can be interpreted as the probability of the presence of a peak. The score value of each peak called by CNN-Peaks is determined by the product of its sigmoid activation value and $-{\log }_{10}$ of the *p*-value of the Poisson distribution for that peak (Supplementary text S5). This score can help users assess the significance of a particular peak.

## Results

### Data preparation and creation of labeled data

We downloaded 16 ChIP-seq datasets and one ATAC-seq dataset in BAM format, which were mapped to the human reference sequence. Professional experts examined 3,294 genomic segments that were randomly selected (Table 1). The datasets were assigned to three experts, each dataset being labeled exclusively by one expert. We expect that there is little experts’ bias in the labeling process^8^.

**Table 1:** A list of the 16 ChIP-seq datasets and the ATAC-seq dataset (in BAM format) downloaded from the ENCODE data portal, and the number of genomic segments labeled as ‘peak’ or ‘no-peak’ by professional experts in each dataset.

Cell line	Target	# of genomic segments labeled	Fraction of peaks labeled near RefSeq tss	ENCODE EXPERIMENT Accession
K562	MAX	152	0.74	ENCSR000EFV
K562	H3K9ac	739	0.82	ENCSR000EVZ
K562	POLR2A	33	0.88	ENCSR338QZF
K562	H3K27me3	9	0.33	ENCSR000EWB
K562	GATAD2	60	0.71	ENCSR547LKC
K562	H2AFZ	27	0.65	ENCSR000APC
K562	Control	437	—	ENCSR000AKY
HepG2	POLR2A	34	0.88	ENCSR000EEM
HepG2	H3K4me2	447	0.81	ENCSR000AMC
HepG2	H3K27me3	15	0.36	ENCSR000AOL
HepG2	H3K9ac	103	0.88	ENCSR000AMD
HepG2	SMARCE1	67	0.78	ENCSR968QDP
A549	H2AFZ	572	0.81	ENCSR000AUH
A549	H3K9me3	87	0.55	ENCSR775TAI
HEK293	H3K9me3	127	0.33	ENCSR000FCJ
GM12878	GATAD2	54	0.71	ENCSR828NCB
A549	ATAC-Seq	297	0.81	ENCSR220ASC
In total, 3294 segments				

Part of these data were labeled and used as training data for our model (note that to put CNN-Peaks through a tough test, our test data does not come from Table 1, but rather from completely different datasets). Our data includes ChIP-seq data for identifying histone modification sites, such as H3K4me3 and H3K27ac, which are known as strong cancer biomarkers^34^. On average, about 66% of the peaks labeled by the experts are near the transcription start sites (TSS) of RefSeq genes, as illustrated in the 4^th^ column of Table 1. This fraction varies from 33% to 88% depending on experiments. We expect that ChIP-seq for H3K9ac histone modification target has a large number of peaks nearby promoters whereas H3K9me3 has much fewer peaks nearby TSS^35^. The labels of ‘peak’ or ‘no-peak’ were applied while visualizing the raw read alignments in BAM format, together with the RefSeq annotation data obtained from the UCSC genome browser. The labeled data were easily created using our own graphical interface program included in the CNN-Peaks software package.

### CNN-Peaks pipeline

**Figure 4:**
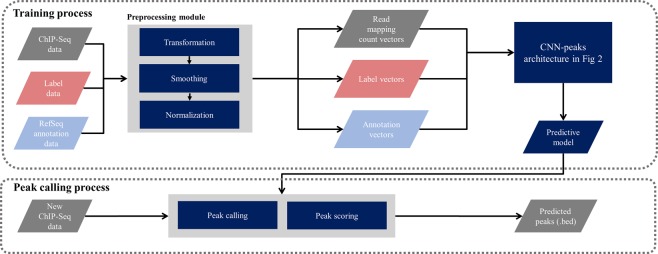
Overview of the CNN-Peaks pipeline. Each parallelogram represents data, a rectangular box an individual module, a rounded rectangle a model trained by CNN-Peaks, and arrows data flow.

We developed the CNN-Peaks pipeline as described in Fig. 4. The CNN-Peaks pipeline takes as training input: labeled data in text format; read alignment data in BAM format; and RefSeq annotation information. The input data is converted to vectors of the right shapes for our CNN architecture. CNN-Peaks trains a predictive model based on the data in these vectors. The model is then applied to unlabeled data in the prediction stage. Our trained model can be used to call peaks for narrow histone modifications and transcription factor binding sites in other human ChIP-seq data (i.e. other than the one in Table 1, part of which was used for training) without additional labeling and training. In addition, peaks in ATAC-seq can also be detected using CNN-Peaks.

Unlike most other peak calling methods, CNN-Peaks needs no additional control ChIP-seq data, usually used as a background signal to reduce false-positive errors. In other words, users can determine the peaks of their target ChIP-seq data with no control data. This is critical when peaks are being called for high-throughput sequencing (HTS) data, such as ATAC-seq and DNase-seq, to capture open chromatin regions, because for the HTS data it is almost impossible to make the control dataset (see the information of ATAC-seq in https://informatics.fas.harvard.edu/atac-seq-guidelines.html).

All modules in CNN-Peaks can be run using a single command. Its output, the peaks called out by CNN-Peaks, is provided in BED format. All of our source code is available in a GitHub repository (http://github.com/odb9402/CNNPeaks). The CNN-Peaks pipeline can be easily installed with Docker, which avoids users having to manually install all prerequisite programs and set up complicated system environments. The graphical interface program for labeling peaks in a given ChIP-seq BAM file is also included in CNN-Peaks. Experienced users can use CNN-Peaks to build a new predictive model, learned from their own labeled data.

### Optimization of hyperparameters in the CNN architecture

There are several hyper-parameters in our package, as listed in (Supplementary text S2). We manually fine-tuned its various hyperparameters, such as the learning rate and convolution filter feature numbers, although we observe that CNN-peak’s performance is not very sensitive to their values. Our CNN architecture uses the Adam Optimizer in TensorFlow-GPU 1.8.0^36^. We trained a predictive model using our CNN-Peaks package with a single Nvidia Quadro P400 GPU within an hour. We used a random subset of all 3,294 genomic segments (see Section 3.1) to train the CNN-peaks model.

### Evaluation using labeled data

Our labeled data in Table 1 were randomly sampled (90%) to build our training set. ChIP-seq data for peaks labeled in (i) the H3K4me3 histone modification in the K562 human leukemia cell line, and (ii) the H3K27ac histone modification in the GM12878 cell line, *were not used* for training the predictive model (note that professional experts marked 156 labels for the H3K4me3 data in K562 and 150 for H3K27ac in GM12878). To evaluate our CNN-Peaks prediction model, we used (i) and (ii) as test datasets, comparing prediction results using CNN-Peaks with the labels in (i) and (ii). We counted false-positive and false-negative errors, and measured sensitivity and specificity. To account for both sensitivity and specificity, we also calculated the F1 score for performance evaluation.

We compared our CNN-Peaks with widly-used peak calling tools, including MACS2, HOMER, and SICER. We used default parameters when running these software tools. There are peak calling results available at the ENCODE data portal for our test datasets (i) and (ii), so we included these results in our performance comparison as well. Note that the peak calling results from ENCODE were processed using an ENCODE ChIP-seq pipeline that used MACS2, as well as post-processing steps to reduce false-positive errors.

**Figure 5:**
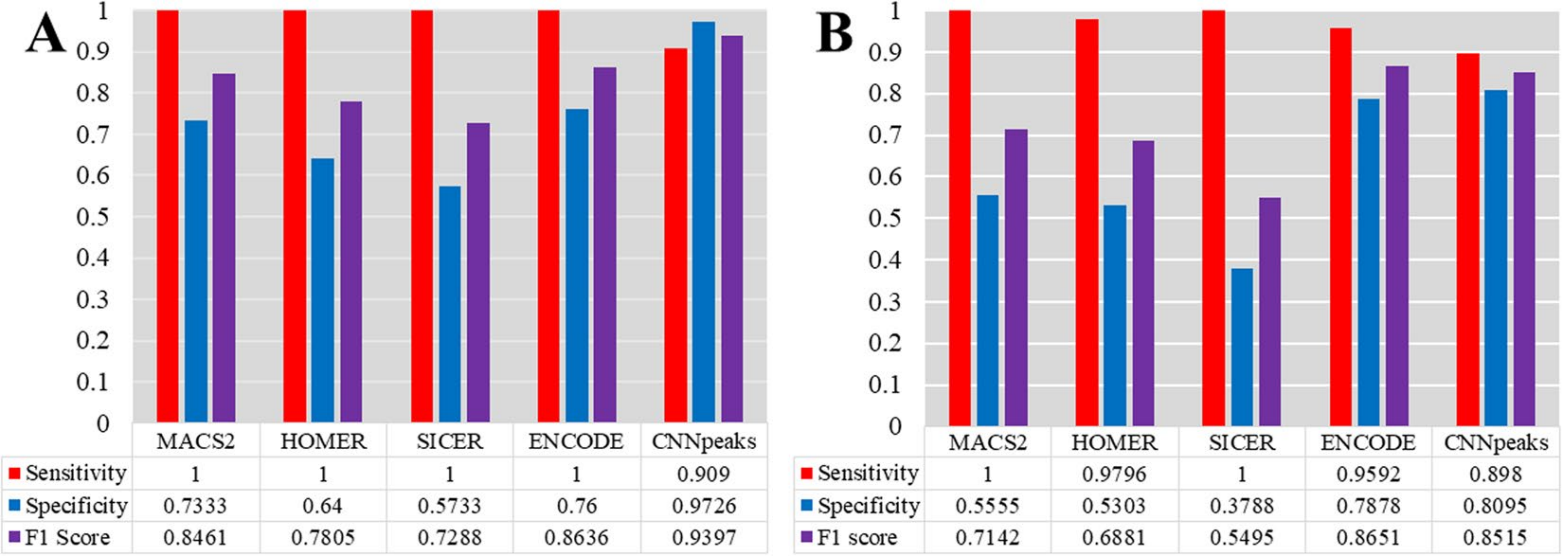
Performance comparison of CNN-Peaks to major ChIP-seq peak calling tools using our labeled testing datasets for (**A**) H3K27ac3 histone modification of GM12878 cell line, and (**B**) H3K4me3 histone modification of K562 cell line. Each blue bar represents sensitivity, and the orange bars specificity. Purple bars show the F1 scores of each peak calling software.

Figure 5 shows the performance comparison for both test datasets. For the GM12878 cell line data (See Fig. 5A), the sensitivity of CNN-Peaks dropped by about 10% compared to the other tools. However, CNN-Peaks improved specificity drastically to almost 97%, while the specificity of the other methods was lower than 76%. As a result, our CNN-Peaks increased the F1 score by at least 8% compared to existing tools. Unlike the GM12878 cell line that is known to have a relatively normal karyotype, the genome of the K562 cell line has more complex karyotypes due to frequent abnormal structural variations^13^. For this reason, calling peaks in the K562 cell line data is more challenging than in the GM12878 data. While the sensitivity of CNN-Peaks dropped by almost 10% for the K562 ChIP-seq data, the specificity increased by 24% compared to the average of the other tools. (see Fig. 5B). In addition to the sensitivity and specificity, we measured the portion of peaks overlapped by the CNN-Peaks and the other three peak callers on the H3K4me3 histone modification ChIP-seq data in the K562 cell line. We observed that 97%, 94%, and 96% of peaks called by the CNN-Peaks overlapped with MACS2, SICER, and HOMER (see Supplementary text S7). Most peaks called by the other peak callers were captured by CNN-Peaks, while many of the false-positive peaks generated by all the three peak callers were filtered out by CNN-Peaks.

We also used the peaks overlapped by all combinations of MACS2, SICER, and HOMER as new predictors, and compared the predictors with the CNN-Peaks. As a result, the intersection of all the three callers showed the highest F1-score (0.7941) for test dataset (i) among all the combinations, but its performance is still not better than that of CNN-Peaks (F1-score 0.8515). In addition, for test dataset (ii), the intersection also showed the highest F1-score (0.8462), but its performance is still not better than CNN-Peaks (F1-score 0.9397). The intersection of peaks called by all the three peak callers may filter out some false-positive peaks, but still contain more false-positive results than our CNN-Peaks.

### Measuring relative distances between narrow histone modification peaks and transcription start sites

Histone modification H3K4me3 and H3K9ac are commonly used as epigenetic markers. The histone modification markers are highly enriched near transcription start sites (TSS), so the relative distances between histone modification peaks and TSS are used for evaluating ChIP-seq peak callers^37,38^. To validate the performance of CNN-Peaks for calling narrow histone modification peaks in the H3K4me3 data of the HepG2 cell line, and H3K9ac of GM12878, we measured the relative distances of our peak intervals and TSS using a similarity metric suggested in^39^ (See Supplementary text S4).

**Figure 6:**
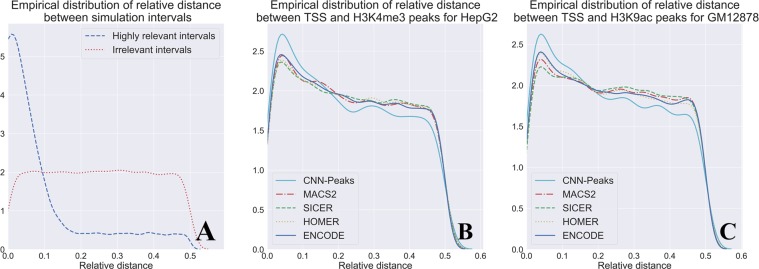
Relative distances between histone modification ChIP-Seq peak calling results and TSS. (**A**) The ideal distribution of relative distances between highly correlated intervals versus the distribution in intervals with no correlation. (**B**) Relative distances between peak intervals and TSS in H3K4me3 data for the HepG2 cell line. (**C**) Relative distances between peak intervals and TSS in H3K9ac data for the GM12878 cell line.

Figure 6A shows the ideal shape for the empirical distribution of relative distances between highly correlated intervals. Figure 6B,C describe the empirical distributions of relative distances between TSS and peaks called by CNN-Peaks, MACS2, SCIER, and HOMER for the H3K4me3 and H3K9ac histone modification data. The plots for CNN-Peaks are more similar to the plots in the ideal case, shown in Fig. 6A, than the plots of other peak calling tools in both datasets. In particular, CNN-Peaks’ plots exhibit an accentuated *y*-value for small relative distances (*x*-value) that then drops. While other tools exhibit a similar behavior, they do not do so as markedly. This indicates that the peaks determined by our CNN-Peaks are more related to TSS than the results of other major tools.

### Peak calling for curated ChIP-seq benchmark data

In addition to histone modification ChIP-seq data, we validate our CNN-Peaks pipeline with other benchmark data that are popularly used for validating software tools of transcription factor binding ChIP-seq analysis^16^. Unlike the data labeled from Table 1 labeled by our experts for this study, this manually curated benchmark data have three classes of labels instead of just the classes ‘peak’ and ‘no-peak’. To be specific, they have an additional ‘ambiguous’ class to mark genomic segments that could not be labeled as either ‘peak’ or ‘no-peak’. In our experiments, we take the ‘ambiguous’ class as ‘no-peak’, which follows the standard of performance measurement for peak calling tools using ChIP-seq benchmark datasets^16^.

We applied our CNN-Peaks, trained with some of our own labeled data from Table 1, to predict the benchmark data whose peaks were determined by manual curation in^16^. We compare our results with the ones predicted by three peak calling programs, including MACS2, HOMER, and SICER. We measure the sensitivity, specificity, and F1 scores using the results predicted by each method and the peaks of the benchmark data in^16^ as ground truth. We ran these three peak calling tools with their default parameters. The whole alignments of all of the ChIP-seq data relative to the reference sequence were fed as their input. While the sensitivity of CNN-Peaks is almost similarly, or slightly smaller, than MACS2, which showed the best sensitivity among the three existing tools, CNN-Peaks’ specificity is the best among the three existing tools, in both the ChIP-seq experiment data for NRSF and SRF targets. Our CNN-Peaks pipeline has substantially better F1 scores than the other tools.

### Validation of peak scoring using known binding motifs

**Figure 7:**
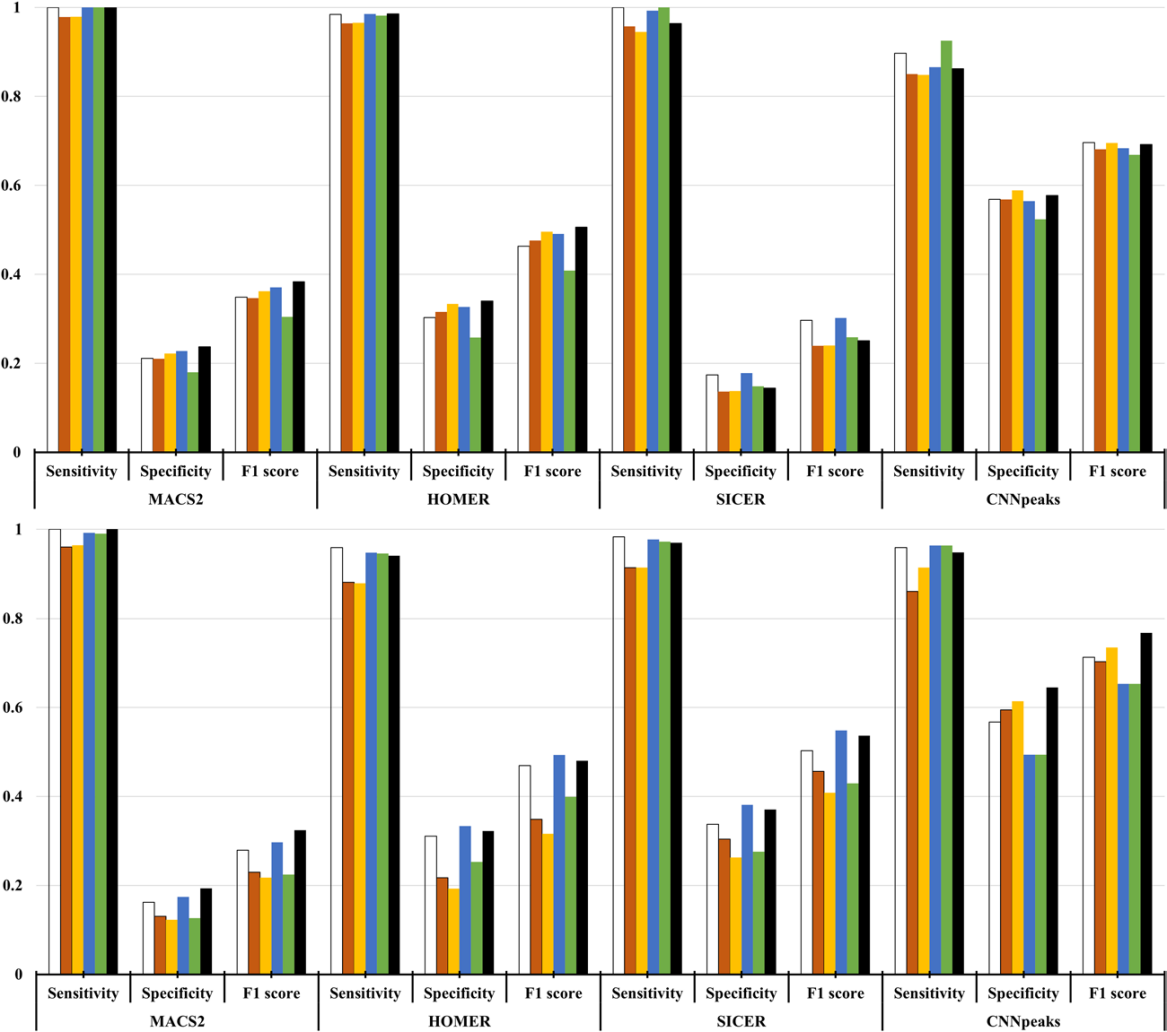
Peak calling performance evaluation using ChIP-seq benchmark data manually curated for SRF target with GM12878 cell line (top) and NRSF target with K562 cell line (bottom). The benchmark dataset includes 6 different types of datasets for the SRF target and NRSF target respectively. These datasets were generated using different methods as described in [16]. Each color represents a different dataset.

**Figure 8:**
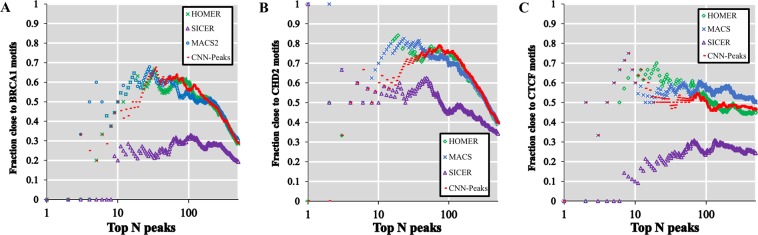
(**A**) Fraction of top N peaks within BRCA1 motif from the GM12878 cell line, (**B**) within CHD2 motif from K562 cell line and (**C**) within CTCF motif from the HepG2 cell line. Top N peaks were determined by scoring peaks called by each software. Each plot shows motif enrichment from transcription factor ChIP-seq data for BRCA1, CHD2, and CTCF respectively.

Comparing the peaks with statistically significant scores (see Section 2.6) for transcription factor ChIP-seq to known binding motifs is another way to evaluate the performance of ChIP-seq peak calling methods^31,40^. We matched peaks determined by four methods, including our CNN-Peaks, to the known binding motifs for several cell lines from ENCODE, including BRCA1 in GM12878, CHD2 in K562, and CTCF in HepG2 (Fig. 8)^40^. In this analysis, we determine the genomic position with the highest score as the center of each peak region, and examine only motif matches close to the center of the peak (*i.e*. within 100 bases around the center of each peak). The goal of this approach is to avoid the analysis to be affected by bias introduced because of the different peak lengths. Users can refine the peak regions determined by CNN-Peaks for the transcription binding ChIP-seq data using a script included in our CNN-Peaks package. We note that for the transcription factor binding ChIP-seq data, CNN-peaks consistently calls peaks wider than MACS2 and HOMER data (see Supplementary text 8). SICER seems to always call peaks wider than any other tool. We use the matchPWM function in the “Biostrings” R package to find binding sites of BRCA1, CHD2, and CTCF^41^. This experiment shows a biological relationship between peak significant scores and actual functional genomics. Figure 8 shows that the scoring system of CNN-Peaks, which uses both the sigmoid activation from the deep learning model and a Poisson distribution, is stable and appropriate. Figure 8 also shows that our CNN-Peaks results are strongly related to binding motifs, and are almost similar to, or better than, the other three peak calling tools. Figures 7 and 8 demonstrate that our trained model that was learned using labeled data is quite robust and generalizes well. Indeed, only 12% of total labels came from transcription factor ChIP-seq, and the rest from histone modification ChIP-seq and ATAC-seq.

## Disscussion

As sequencing has become readily available for biological and medical studies, GWAS studies have identified many DNA bases and structural variations linked not only to genetic diseases but also to common diseases. While GWAS suggests what DNA abnormalities are linked to diseases, such studies cannot characterize the functional aspects of such variations. ChIP-seq has been widely used to understand the mechanisms of genome-wide gene regulation involved in developmental biology and various diseases, including cancer. Many computational approaches have been developed and applied to analyze the ChIP-seq data. However, most of them exhibit a high false-positive ratio of peak signals, and can misinterpret data, especially for histone modification ChIP-seq data.

To resolve this issue, we designed a new approach for calling the peaks in ChIP-seq data based on a convolutional neural network architecture that mimics human visual inspection. Our tool uses genome annotation information, labeled data for peaks that are inspected by human researchers, as well as read mapping data in BAM format for ChIP-seq data. Our ChIP-seq data were generated from GM12878 and K565 human cell lines. Our CNN prediction method, which comes integrated with some useful data preprocessing steps, is available as a software package called CNN-Peaks. This package also includes a graphical visualization interface for users to easily set labels ‘peak’ or ‘no-peak’ in genomic segments using read mapping BAM files.

We tested our predictive model and reported test errors using both our own labeled data, and benchmark data that were manually curated by other groups, and that are popularly used to evaluate computational methods for calling peaks. Our CNN-Peaks showed a quite conservative peak calling rate and improved specificity compared to other methods, while not dropping sensitivity much. Hocking *et al*. (2017) first proposed the idea of using visual inspection for calling peaks, and showed that manual peak calling could improve the performance of existing peak calling tools. However, they only focused on tuning parameters of existing peak callers using labeled data, while we redefined this idea as a signal processing problem, and designed a new approach based on the convolutional neural network model, which has been successfully used in various signal processing problems. Two important details contribute to its improved filtering out of false-positive peaks: its use of *inception modules* and its use of additional information, such as RefSeq annotation from experts’ visual. These factors increase the specificity and F1 scores of CNN-Peaks’ prediction comparing to other existing peak calling tools, even for ChIP-seq data that were generated in the K562 cancer cell line, which is known to be extremely complex due to frequent abnormal structural variations. In addition, we investigated the biological relationship between peak calling results and transcription start site (TSS) for histone ChIP-seq, and with known binding motifs for transcription factor ChIP-seq. Our experiments demonstrate that the peaks of histone modification ChIP-seq data determined by CNN-Peaks are highly correlated with TSS, and that the peaks of transcription factor ChIP-seq are strongly related to known binding motifs.

There is still some room to improve the performance of our pipeline. CNN-peaks can be improved by collecting more labeled data are collected by more experts (see Supplementary text 6). Non-experts’ opinions can also be used to improve its performance, since their labeling results should be mostly consistent with that of the experts (see Supplementary text 9). We can further include other types of information that professional experts use for inspecting the peaks in ChIP-seq data via our visualization tool. The more thoroughly curated data are available for various types of ChIP-seq datasets, the more we expect that our performance will improve. In addition to calling peaks, we believe that our CNN-Peaks pipeline could be extended to determine if ChIP-seq datasets are problematic or valid. One straighfoward way of doing so is to look at the confidence scores output by CNN-Peaks and determine a dataset to be problematic if most confidence scores are low. This approach is not fail proof, as one could imagine a problematic dataset with very clear (but wrong) peaks. One way to tackle this, would be to ask experts not only to label peaks, but also to label the correctness of different regions in the training data. A CNN could then be trained to minimized a combined loss for (a) predicting the occurences of peaks, and (b) determining the correctness of different regions in the input data. CNN-Peaks can be a useful toolset for the quality control and accurate analyses of high throughput sequencing data in the epigenetic research community.

## Supplementary information

Supplementary information.
