# Supplementary material for: CNN-Peaks: ChIP-Seq peak detection pipeline using convolutional neural networks that imitate human visual inspection
Source: Sci Rep. 2020 May 13;10:7933. doi: 10.1038/s41598-020-64655-4 (PMC7220942; doi:10.1038/s41598-020-64655-4)
Supplement: Supplementary file 1 — Supplementary information. [file 41598_2020_64655_MOESM1_ESM.docx]

Supplementary Information for “CNN-Peaks: ChIP-Seq peak detection pipeline using convolutional neural networks that imitate human visual inspection”

Dongpin Oh^1^, J. Seth Strattan^2^, Junho Hur^3^, José Bento^4^, Alexander Eckehart Urban^2^, Giltae Song^1,*^, and J. Michael Cherry^2^

^1^School of Computer Science and Engineering, Pusan National University, Busan 46241, South Korea, ^2^Department of Genetics, Stanford University, Stanford 94305, USA, ^3^School of Medicine, Kyung Hee University, Seoul 02447, South Korea, ^4^Department of Computer Science, Boston College, Chestnut Hill, MA 02467, USA

*To whom correspondence should be addressed.

# **Supplementary Text 1.** The preprocessing module

The labeled data are split into multiple windows of various sizes using a density-based clustering approach, the DBSCAN algorithm (Figure S1). To make windows of different sizes fit the CNN model, we normalize their sizes by mapping all of the clusters of various sizes into vectors of the same size, and by sampling from a large window using bins (note that the size is set as 12,000 bins by default and users can adjust it). Through this preprocessing module, the vectors for the read mapping information, the labeled data, and the annotation information are shaped into a common size. The vector for the read mapping data has numeric values that indicate the depth of reads mapped in each individual genomic position compared to the reference sequence, and the vector for the labeled data is composed of binary values that represent the absence, or presence, of peaks.

Figure S1. The schema of the preprocessing module of CNN-peak. (A) a process extracts windows of various sizes and normalizes them to the same width. Black signals in each window expresses read mapping counts, red underlay labels for peaks, and yellow underlays for no peaks. (B) a module converts two input information (read mapping counts and RefSeq annotation) and labeled data for a single window into three vectors that will be input to the CNN. RefSeq (genomic, transcript and protein encoding annotation information from NCBI) is described using a bar filled in blue for an annotated gene and empty bar for no genes, below on the left-hand side. The rhree rows in the right-hand side indicate three vectors of the same size.


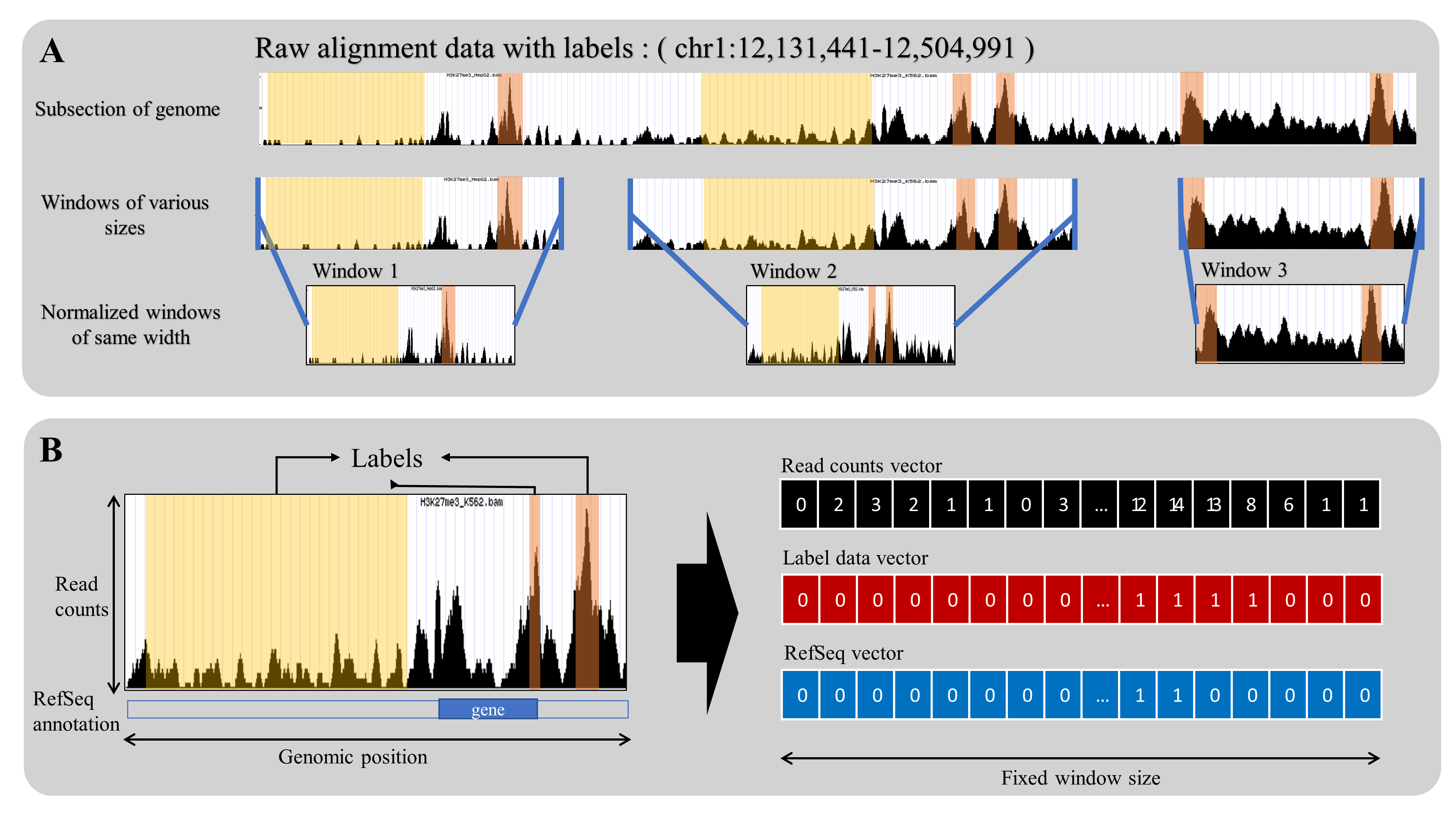


Using this pre-processing module, the raw data of the peak annotations (of the form chrXX:XXXX-XXXX for each peak) is converted into a vector of dimensions that fit the CNN-Peaks model. However, if a window of genomic segments that includes labels is smaller than the target window size for normalization, the user needs to eliminate the window, extend its visual inspection to make the window wider than the target size, or adjust the parameter of the target window size.

**
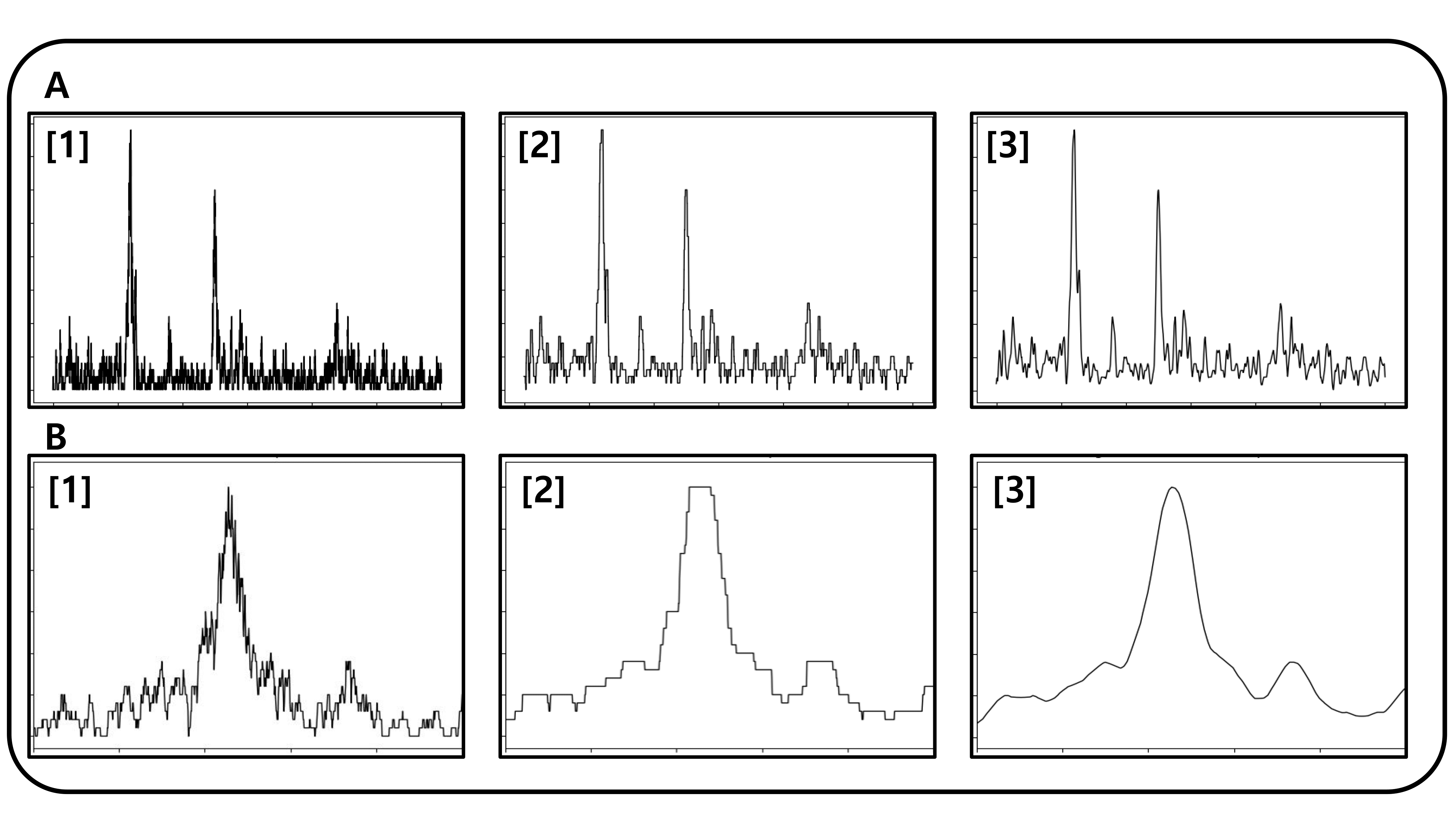
**

Figure S2. Smoothing of ChIP-seq read mapping data. (A) and (B) show read mapping patterns in two different genomic segments. Each row shows: [1] a visualization of initial read mapping data; [2] an intermediate outcome of the smoothing; and [3] the final smoothed mapping patterns.

# **Supplementary Text 2.** Convolutional neural network structure and hyperparameters of CNN-Peaks

**Table S1.** A list of hidden layers in the CNN architecture of the CNN-Peaks package, and the number of parameters in each layer.

| Name | Dimension of output | # of parameters |
| --- | --- | --- |
| Input Read Mapping Counts | (1, 12000, 1) | 0 |
| Input RefSeq | (1, 12000, 1) | 0 |
| Convolution 1 | (1, 12000, 16) | 64 |
| Convolution 2 | (1, 12000, 32) | 1,024 |
| Max pooling | (1, 6000, 32) | 0 |
| Ref Convolution | (1, 12000, 4) | 16 |
| Ref Max pooling | (1, 6000, 4) | 0 |
| Inception (A) – 1 | (1, 3000, 120) | 5,184 |
| Inception (A) - 2 | (1, 1500, 336) | 34,560 |
| Inception (B) - 1 | (1, 500, 480) | 161,280 |
| Inception (B) - 2 | (1, 250, 864) | 552,960 |
| Inception (B) - 3 | (1, 125, 1440) | 1,492,992 |
| Inception (C) - 1 | (1, 25, 2048) | 21,381,120 |
| Inception (C) - 2 | (1, 5, 2880) | 42,762,240 |
| Average pooling | (1, 1, 2880) | 0 |
| Fully Connected1 | (1, 500) | 1,474,560 |
| Fully Connected2 | (1, 250) | 262,144 |
| Output Layer (Threshold) | (1, 10) | 5,120 |
| Output Layer (Expanded) | (1, 12000) | 0 |
| Total number of the parameters: 68,133,264 | | |

When converting the thresholded output layer to the expanded output layer, by subtracting *Output Layer (Threshold)* from *Input read mapping counts*, our CNN uses the TensorFlow broadcast rules, which can deal with operations between data of different dimensions (<https://www.tensorflow.org/xla/broadcasting>). The structure and the hyperparameters described in Supplementary Text 2 were fixed during all of the experiments, including the evaluation with labeled data (histone modification), the evaluation with benchmark data (transcription factors), and the BRCA1 motif analysis. The values for the hyperparameters were set as follows.

- Learning rate: 0.0001 ( For Adam optimizer.)
- Generations ( epochs ) : 1500
- Filter size of max-pooling in preprocessing: 101
- Filter size of Gaussian filter in preprocessing: 301
- # of output threshold: 50
- Mini-batch size: 1 ( Online learning )
- Padding type of max pooling and convolution: “same”
- Type of activation functions : “ReLU”

# **Supplementary Text 3.** Python application for labeling tasks


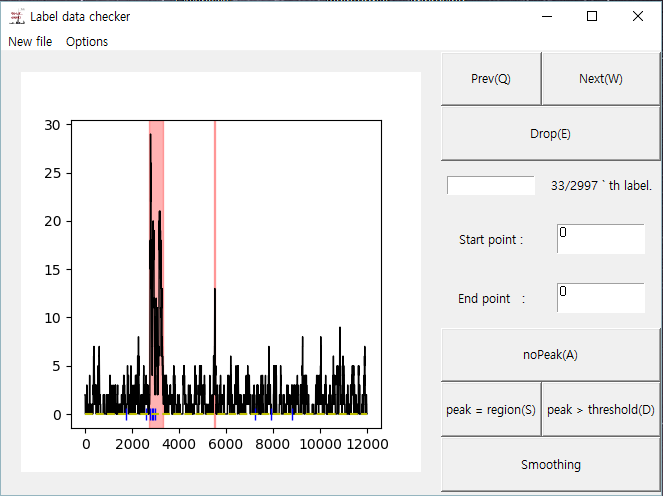

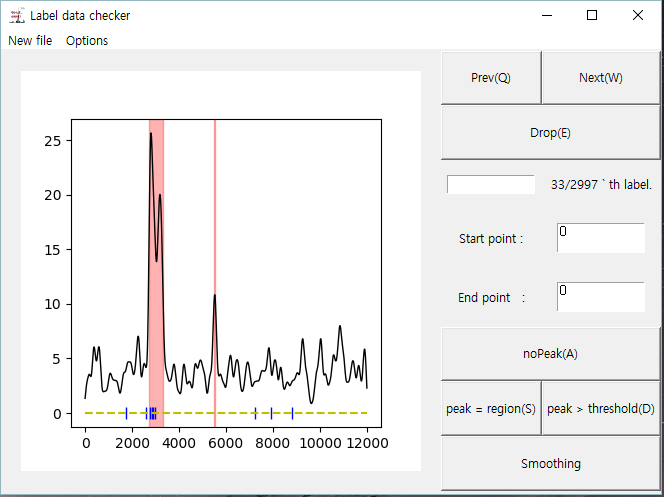


A

B

Figure S3. A graphical user interface (GUI) for labeling peaks is included with the CNN-Peaks package. (A) is the GUI output image for the raw read mapping counts. (B) is the visualization of the smoothed read mapping patterns in our GUI application. The black lines represent read mapping count patterns, and the blues lines the presence of RefSeq annotations. Red highlights are peaks labeled by experts. Yellow lines are a user-defined threshold value for peaks. Users have two ways to generate red highlights that indicate peaks. They can either select a peak region by entering its start and end positions, or they can input a threshold value and choose genomic segments for which read counts are larger than this threshold. These tasks can be done by mouse drag and click, provided by the GUI interface. The black lines (i.e. read mapping patterns) can be also smoothed using extra user-defined settings.

# **Supplementary Text 4.** Relative distance to measure the similarity between two interval sets.


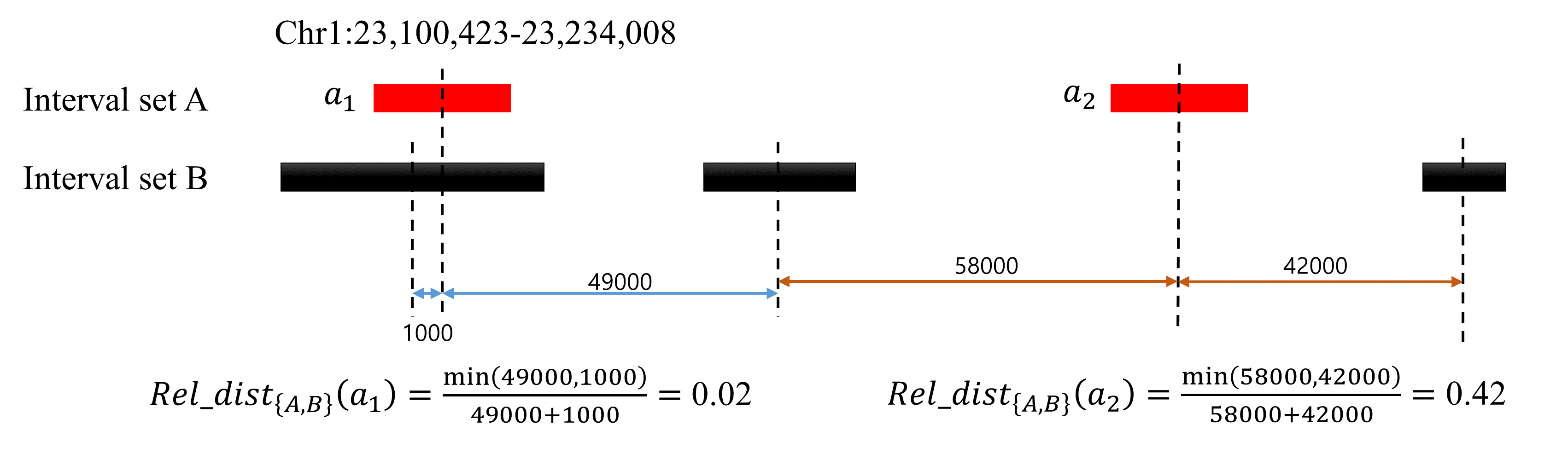


**Figure S4.** The description of relative distance. Variable *a_i_* is the *i*th interval in *A*. For each interval *a_i_*, we compute its center position *x,* and center positions *y* and *z* of the intervals in *B* whose centers are directly next to the right and left of *x*. Then, the relative distance between *A* and *B* for *a_i_,* denotes by *Rel_dist*_{_*_A_*_,_*_B_*_}_(*a_i_*), is min(*y* - *x*,*x* - *z*)/(*y* – *z*). The relative distance can reveal a similarity between intervals using the spatial information of each interval. If *B* has an interval whose center equals the center of *a_i_*, then *Rel_dist*_{_*_A_*_,_*_B_*_}_(*a_i_*) = 0.

# **Supplementary Text 5.** The output format of CNN-Peaks


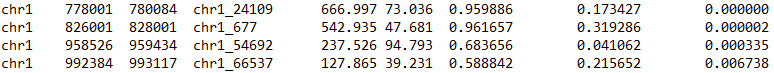


Figure S5. An example of an output file from CNN-Peaks.

The output format of CNN-Peaks follows the standard BED format, which is tab-delimited. The 1^st^ to 5^th^ columns display the same information as the annotation track of the UCSC genome browser and IGV [1][2].

Each row of the CNN-Peaks output includes the following information.

1. **Chromosome** – The name of the chromosome.
2. **Chromosome Start** – The starting position of the features in the chromosome.
3. **Chromosome End** – The ending position of the features in the chromosome.
4. **Name** – A randomly generated name for the peak.
5. **Score** – The score of the peak signal, determined from columns 6 and 9 (Equation [S5]).
6. **Score2** – The score of the peak signal, determined from columns 6 and 8 (Equation [S6]).
7. **Sigmoid activation value** – The sigmoid activation value of the peak, from CNN-Peaks output.
8. **P-value (Avg)** – Average p-value in the peak region, assuming a Poisson distribution for the number of reads in a position.
9. **P-value (Min)** – Minimum p-value in the peak region, assuming a Poisson distribution for the number of reads in a position.

The score in the 5^th^ column is determined via Equation (S5).

|  | $\boldsymbol{100\cdot-lo}\boldsymbol{g}_{\boldsymbol{10}}\left( \boldsymbol{pValu}\boldsymbol{e}_{\boldsymbol{min}} \right)\boldsymbol{\cdot Sigmoid}$ | (S5) |
| --- | --- | --- |

Above, $pValue_{min}$ is the minimum p-value in the peak region, assuming a Poisson distribution, (9^th^ column), and $Sigmoid$ is the sigmoid activation value of the peak (6^th^ column).

The score in the 6^th^ column is determined via Equation (S6).

|  | $\boldsymbol{100\cdot-lo}\boldsymbol{g}_{\boldsymbol{10}}\left( \boldsymbol{pValu}\boldsymbol{e}_{\boldsymbol{avg}} \right)\boldsymbol{\cdot Sigmoid}$ | (S6) |
| --- | --- | --- |

Above, $pValue_{min}$ *is* the average p-value in the peak region, assuming a Poisson distribution, (8^th^ column), and $Sigmoid$ **is the** sigmoid activation value of the peak (6^th^ column).

# **Supplementary Text 6.** The effect of the training dataset size

To see the effect of training data size on performance, we trained our model using different training data sizes and evaluated its performance on a test dataset that was obtained also from sampling segments from Table I in the main document. Notice that this test dataset is different from the test dataset used in Figure 5 in the main document. We used four training datasets of sizes 1647, 1098, 549, and 336 as the training data for CNN-Peaks. For each training dataset, we used the remaining segments in Table 1 as the test set. Figure S6 shows the F1 scores for training and testing in each of the four datasets. The F1 scores on the test set (i.e. the maximum of each blue curve) do not drop for training datasets of sizes 1647 and 1098, but start decreasing for size 549, as the size of the training data decreases. This means that the 3,294 genomic segments are sufficient as training data. The performace could still improve a bit, probably not by much, if more training datasets were used.


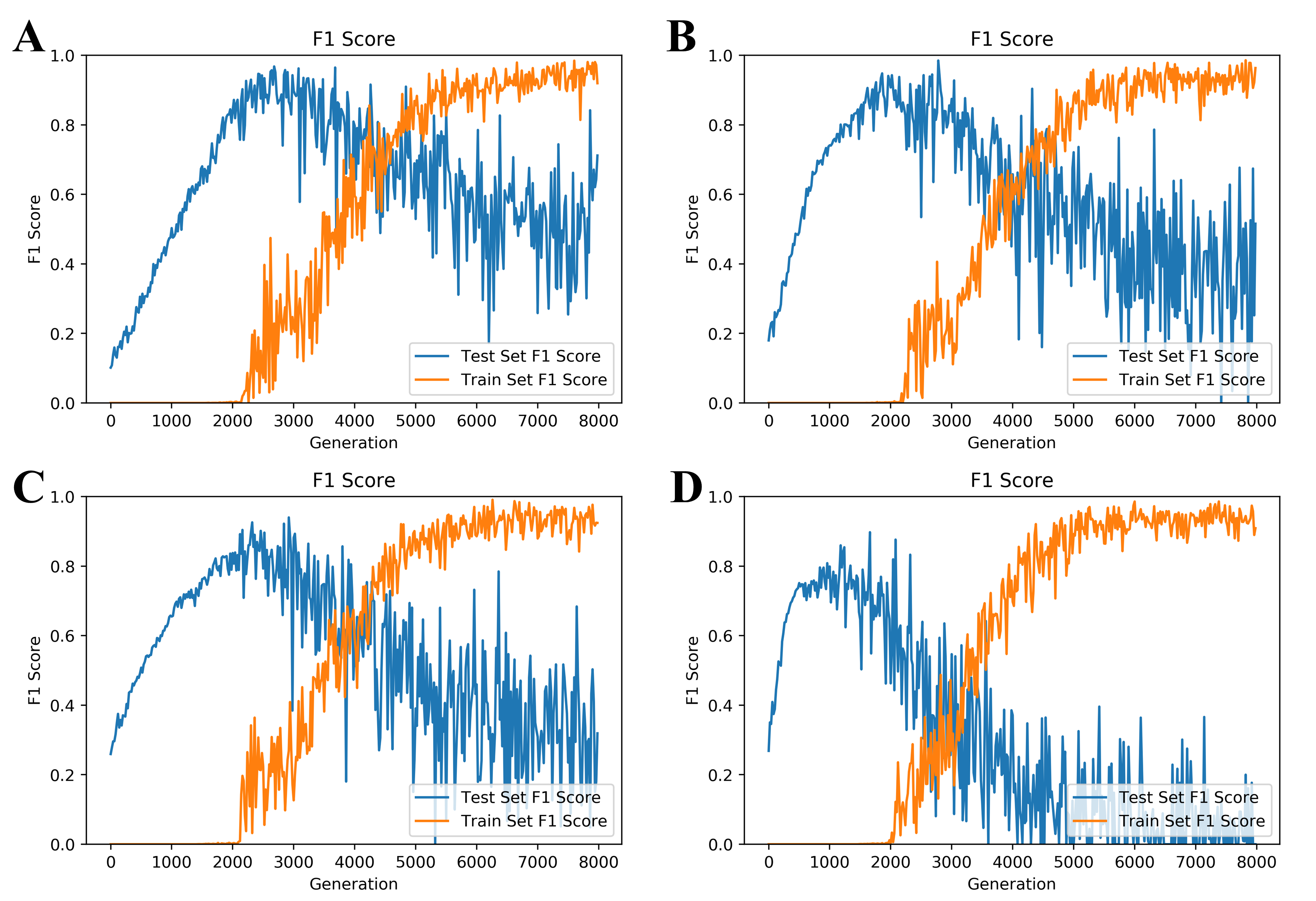


Figure S6. The learning curve for F1 Score of CNN-Peaks training processes. Blue lines are the F1 Score of test data (separated data) during the training processes. Yellow lines are the F1 Score of training data during the training processes. A) The learning curve using 1647 segments. B) The learning curve using 1098 segments. C) The learning curve using 549 segments. D) The learning curve using 336 segments. Our experience shows that the F1 score on the test data during training should be over 0.9 to get good performance on new datasets, so we do not recommend using sizes below that C) and D) to train the CNN-Peaks model.

# **Supplementary Text 7.** Peak overlaps among different peak-calling algorithms

We examined the overlaps of called peak intervals between CNN-Peaks and the other three peak callers on the H3K4me3 histone modification data for the K562 cell line.


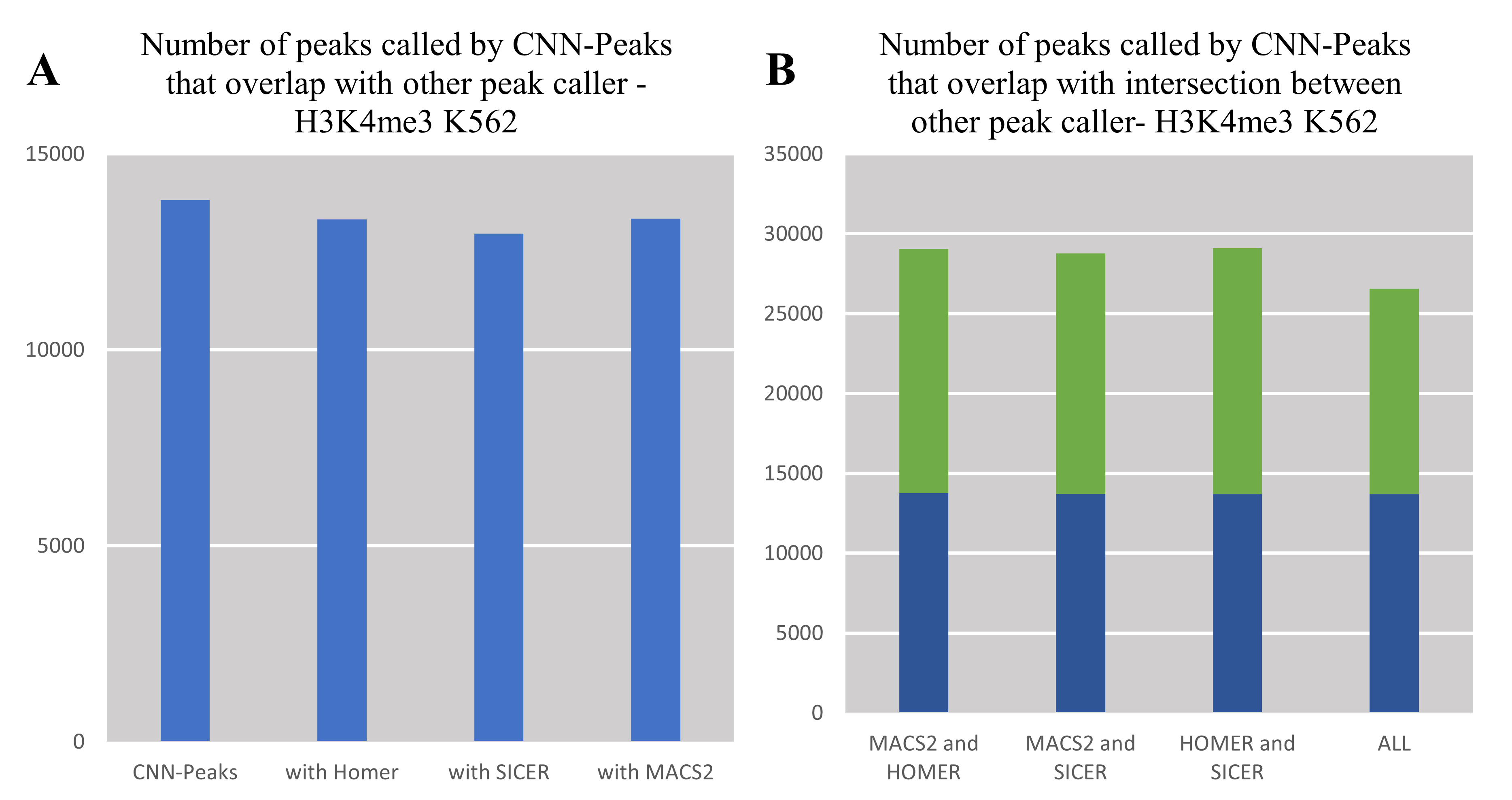


Figure S7. The number of peaks generated by CNN-Peaks, which overlap with the three other peak callers for the H3K4me3 histone modification ChIP-seq with H3K4me3 cell line. A) The number of peaks called by CNN-Peaks, which overlap with the three peak callers. The first column is the total number of peaks called by CNN-Peaks. B) The number of peaks called by CNN-Peaks, which overlap with the intersection of the results generated by other methods. The blue bars represent the number of peaks that match with the CNN-Peaks results. The green bars represent the number of peaks that overlap among the group of other tools being considered.

# **Supplementary Text 8.** Interval length of the peaks from CNN-Peaks

The length of the intervals called by SICER is in general much larger than the lengths called by the other tools. HOMER and MACS2 typically call shorter intervals than the other tools. CNN-Peaks’ interval lengths seem to be a middle ground between these two extremes. Notice that we expect the interval lengths in Table S3 to be in general much larger than the interval lengths in Table S4 across all tools.

**Table S3.** The average interval length of CNN-Peaks and other peak calling software for the histone modification ChIP-Seq on four different datasets.

|  | H3K4me3  K562 | H3K27ac GM12878 | H3K4me3 HepG2 | H3K9ac GM12878 |
| --- | --- | --- | --- | --- |
| CNN-Peaks | 1994 | 1963 | 2253 | 2204 |
| HOMER | 1779 | 2124 | 1786 | 1856 |
| SICER | 4086 | 3584 | 2818 | 4035 |
| MACS2 | 1659 | 2206 | 1630 | 1919 |

**Table S4.** The average interval size of CNN-Peaks and other peak calling software for the transcription factor binding ChIP-Seq on another set of four different datasets.

|  | NRSF  K562 | SRF GM12878 | CTCF HEK293 | BRCA1 GM12878 | CHD2 GM12878 |
| --- | --- | --- | --- | --- | --- |
| CNN-Peaks | 1428 | 1267 | 1420 | 1099 | 1142 |
| HOMER | 163 | 152 | 169 | 140 | 184 |
| SICER | 4173 | 4372 | 1084 | 4263 | 3749 |
| MACS2 | 176 | 150 | 211 | 223 | 290 |

# **Supplementary Text 9**. Labeling results by two non-experts

Labeled data for an ENCODE H3K9ac histone modification ChIP-seq data with a B-cell cell line was generated by two non-experts, using our labeling application in Supplementary Text 3 (ENCODE accession: ENCSR799SLA). The non-experts only trained for the usage of the labeling application and the patterns of ChIP-seq alignments. The 50 curated genomic segments were created in approximately 10 minutes by each curator.


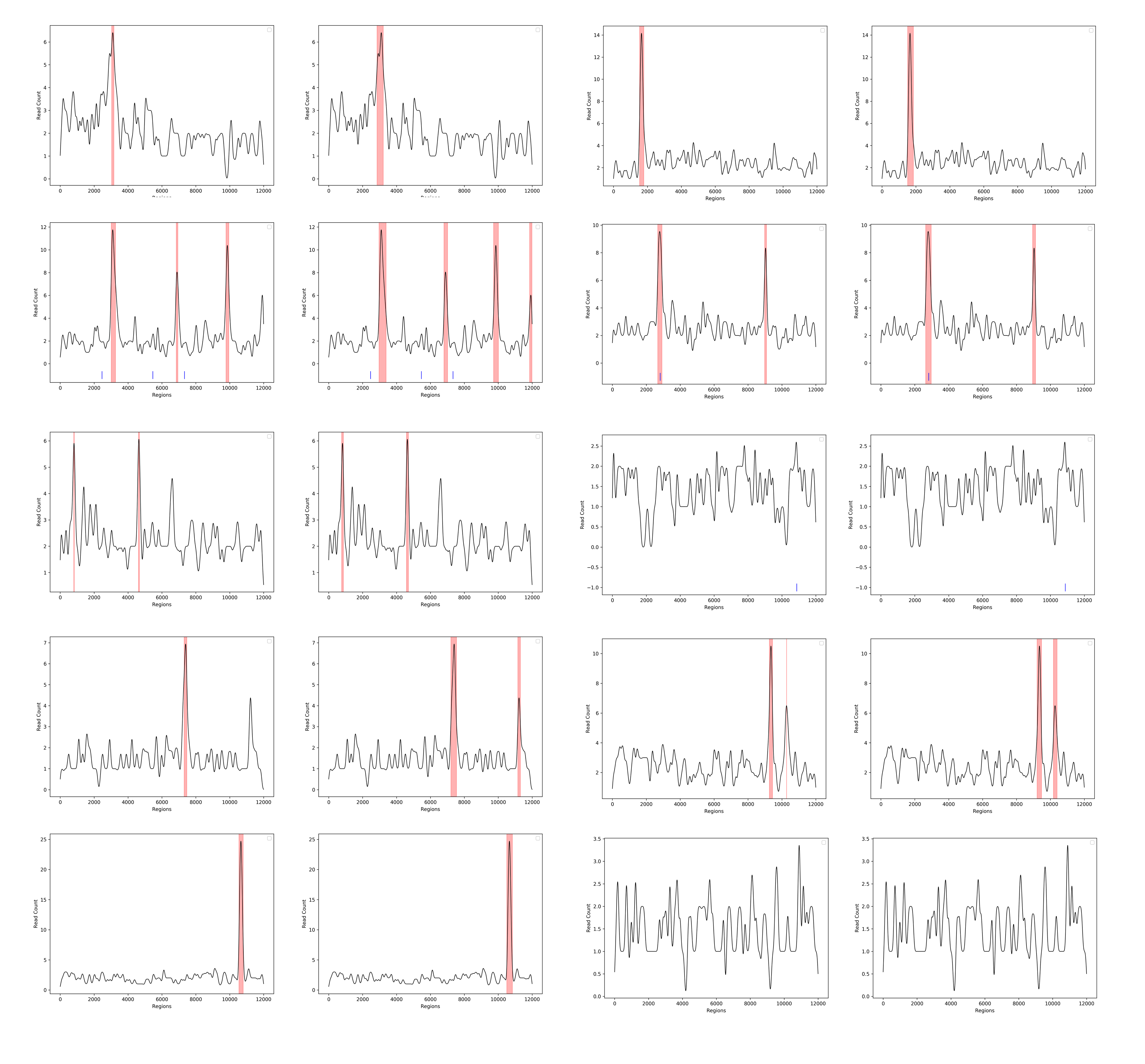


Figure S7. Examples of genomic segments labeled by non-experts. The red highlights illustrate peak regions. The black lines illustrate smoothed ChIP-seq alignment patterns. The 1^st^ and 3^rd^ columns were generated by one non-expert. The 2^nd^ and 4^th^ columns were generated by another non-expert. The peak results called by these two non-experts were almost identical (the only exception being the first two plots in the third row above). These results were also consistent with our expert’s peak calling.

# **References**

[1] Kent, W. J. *et al.* (2002). The human genome browser at UCSC. *Genome Research*, *12*(6), 996-1006.

[2] Thorvaldsdóttir, H. *et al.* (2013). Integrative Genomics Viewer (IGV): high-performance genomics data visualization and exploration. *Briefings in bioinformatics*, *14*(2), 178-192.
